# Neonatal mortality and associated factors among neonates admitted to neonatal intensive care unit of Gandhi memorial hospital in Addis Ababa, Ethiopia, 2019

**DOI:** 10.1186/s12887-022-03339-6

**Published:** 2022-05-12

**Authors:** Metasebiya Zelalem Ayichew, Lemma Derseh Gezie, Abebaw Addis Gelagay, Desalegn Anmut Bitew

**Affiliations:** 1 Gandhi Memorial Hospital, Addis Ababa, Ethiopia; 2 Department of Epidemiology and Biostatistics, Institute of Public Health, College of Medicine and Health Sciences, University of Gondar, Po. Box 196, Gondar, Ethiopia; 3 Department of Reproductive Health, Institute of Public Health, College of Medicine and Health Sciences, University of Gondar, Po. Box 196, Gondar, Ethiopia

**Keywords:** Neonatal mortality, Neonates, Neonatal intensive care unit, Gandhi memorial hospital

## Abstract

**Background:**

Ethiopia witnessed an unprecedented decline in under-5 and neonatal mortalities since 2000. But, neonatal mortality still accounts for the largest proportion of under-five child mortality. Quality of service at hospitals may vary and determine the magnitude of neonatal mortality.

**Objective:**

To assess the prevalence and associated factors of neonatal mortality among newborns Admitted to the Neonatal intensive care unit of Gandhi Memorial Hospital Addis Ababa, Ethiopia, 2019.

**Methods:**

Institution-based cross-sectional study was conducted from November 1 to December 31, 2019. A sample of one in every 2 admitted patients was included in the study. our exclusion criterion was neonates who had no mothers or guardians and/or neonatal medical records incomplete for status at discharge. We used the Systematic random sampling technique to select the study participants. A pretested structured interviewer-administered questionnaire and a preliminary tested checklist were used to collect primary and secondary data respectively. Descriptive and summary statistics were performed. A binary logistic regression model was fitted and variables that had a *P*-value of < 0.05 in the multivariable model were considered statistically significant.

**Results:**

A total of 570 neonates who have mothers were involved in the study giving a response rate of 98.8%. The prevalence of neonatal mortality was 7.7% (95% CI: 5.7, 9.8). Mothers' educational status (No education (AOR 3.37, CI 95%, 1.02–11.20), premature rupture of membrane (prolonged PROM) (AOR 5.59, CI 95%, 1.05–29.76), and birth weight less than 2500gm (AOR 3.23, CI 95%, 1.17–8.90) are the significant factors associated with neonatal mortality.

**Conclusion:**

The prevalence of neonatal mortality at Gandhi memorial hospital was generally high. As our finding revealed, neonates who are underweight and whose mothers have no formal education as well as have prolonged PROM have higher odds of neonatal mortality. Thus, clinicians, policymakers, and program managers should give special attention to neonates of none educated mothers, mothers with prolonged PROM, and neonates with low birth weight.

**Supplementary Information:**

The online version contains supplementary material available at 10.1186/s12887-022-03339-6.

## Introduction

The neonatal period, which incorporates the first 28 days of the infant’s life, is the most crucial time for a child’s survival and as it accounts for nearly half of the deaths of children below five years of age [1]. The neonatal mortality rate (NMR) is an important metric for measuring a society's overall quality of life. NMRs are frequently used as a standard index for evaluating health, education, and social systems, as well as nutritional status and health programs for newborns in countries, and identifying the causes of mortality is the first step in lowering mortality rates [2].

Although global neonatal mortality has dropped significantly from 5 million in 1990 to 2.4 million in 2019, newborn death still accounts for 47% of all deaths among children under the age of five, with nearly one-third dying on the day of birth and nearly three-quarters dying within the first week of life [3]. Each year, around 3.1 million babies die in their first month of life around the world, with the bulk of these deaths occurring in underdeveloped countries [4]. More than 1.1 million newborn fatalities occur in Sub-Saharan Africa, accounting for 28% of the global burden. Nigeria, Ethiopia, the Democratic Republic of the Congo, and Tanzania each contribute 6%, 4%, 3%, and 2% of the global burden of neonatal mortality, respectively [5]. Despite improvements over the past decade, according to the 2016 Ethiopian demographic health survey (EDHS) report, the neonatal mortality rate was 29 deaths per 1,000 live births which is still one of the highest in the world [6].

Perinatal death is related to problems such as intrauterine growth restriction, embryonic asphyxia (such as fetal immaturity), severe congenital malformations, early infection and neonatal risk, low birth weight, premature birth, and fatal congenital abnormalities [1, 7]. Some local studies conducted in Ethiopia have also reported that sepsis, asphyxia, birth injuries, tetanus, preterm delivery, and congenital malformations are primarily associated with neonatal mortality [8].

As to the best knowledge of the researcher, neonatal mortality and its associated factors in the hospital are not studied and are not well documented mainly at the institutional level including in this study area, Gandhi Memorial Hospital. Therefore, the purpose of this study was to assess neonatal mortality and associated factors among neonates admitted to the neonatal intensive care unit (NICU) of Gandhi memorial hospital.

## Methods

### Study design, study setting, and study period

An institution-based cross-sectional study was conducted at Gandhi memorial hospital, Addis Ababa, Ethiopia from 1^st^ November to 31 December 2019. There are 12 governmental Hospitals in Addis Ababa, of these only six hospitals (Black Lion, St. Paul, Gandhi, Zewditu, Yekatit 12, and Tirunesh Beijing) have their own neonatal intensive care unit (NICU). Gandhi Memorial hospital is a governmental hospital that specializes in maternity services. The hospital serves as a teaching center for Addis Ababa University students who specialize in gynecology and obstetrics and other undergraduate medical students. The hospital daily manages 40–50 delivery cases of pregnant mothers who come from various corners of Addis Ababa and nearby towns.

The hospital NICU receives high-risk babies delivered within the institution, referrals from other health facilities, and referrals from home deliveries. On average the hospital has 6,658 annual and 555 monthly admissions to NICU. The NICU had a 32-bed capacity and was staffed with two Public health officer professionals, one Neonatologist, and 22 nurses.

### Populations of the study

All neonates, who were admitted to the NICU of Gandhi Memorial Hospital in Addis Ababa, were the source population and all neonates who were admitted to the NICU of Gandhi Memorial during the study period, were the study population.

### Inclusion

A sample of one in every 2 admitted neonates was included in the study.

### Sample size determination

The sample size was determined by using both single and double population proportion formulas for the first and second objectives respectively.

For the single population formula,

We used a 4% margin of error, 95% confidence interval and Prevalence of Neonatal mortality (35.5%) from the previous study conducted in Jimma Zone [9] and the calculated sample size was 549.

By taking additional 5% contingency for non-response rate, 5%*549 = 27.45, therefore the total sample size found to be = 577.

Since the sample size calculated for objective one (*n* = 577) was greater than the sample size calculated for objective 2, the final sample size was 577.

### Sampling technique and procedure

A systematic random sampling technique was used to select participants (neonate's mother) attending the Gandhi Memorial Hospital. The monthly average number of neonates admitted to the Gandhi memorial hospital was estimated by reviewing the hospital logbook for the previous three years and found 600 per month. This gives an average two months admission of 1,200 neonates. Using the two-month admission as the study population, we determine the 'k' value by dividing 1,200 by 577 which gives approximately 2. Therefore, to select the first participant, we randomly select one number from 1 and 2. One was drawn. Then, every other (2^nd^) subject was included until the final sample size is achieved.

### Variables of the study

The dependent variable in this study was neonatal mortality whereas the explanatory variables were socio-demographic and socio-economic factors such as maternal age, maternal education, place of residence, marital status, economic status and religion, reproductive and obstetric factors (complication during pregnancy, parity, ANC, birth order interval, PROM, type (mode) of delivery, TT immunization, gestational age at birth, initiation of breastfeeding, exclusive breastfeeding), health care practice and related factors (place of delivery and skilled birth attendant), neonatal related factors (age at admission, sex of the baby, birth weight of the baby, APGAR score) and medical factors (hypothermia, hypoglycemia, asphyxia, infection, prematurity, congenital malformation, and birth trauma).

### Operational definitions

Neonatal Mortality: Death of neonates within the first 28th day of life.

Congenital Malformations: are structural or functional anomalies that occur during intrauterine life and can be identified prenatally, at birth, or sometimes may only be detected later in infancy [10].

Hypoglycemia: A measure of low blood glucose (< 40 mg/dL) that was diagnosed and recorded on charts by professionals on admission.

Hypothermia: Any low body temperature measurement (< 36 °C) diagnosed and recorded on or during admission of neonates.

Premature birth: Any viable neonate before term (< 37 weeks of gestation) that was already diagnosed by professionals in charge of admission of neonates to neonatal intensive care units [11].

Premature Rupture of Membrane (PROM): Rupture of membrane before the onset of labor. Prolonged rupture of membranes (PROM) is considered when the duration is more than 12 h before delivery [12].

Stillbirth: it was measured as fetal death (i.e., death before the complete expulsion or extraction of a product of conception from its mother) in the third trimester (≥ 28 completed weeks of gestation) using the WHO definition of stillbirth [13].

Abortion: is the termination of a pregnancy that occurs with/without intervention (a miscarriage or "spontaneous abortion" or induced abortion).

### Data collection procedure

A structured questionnaire and checklist were developed based on literature for collecting data from study participants in the Hospitals. The Structured questionnaire and checklist were prepared in the English version and translated to Amharic to maintain smooth communication with participants and translate back to English to see the consistency and clarity of the questionnaire. The medical diagnosis information was collected from the chart of the newborn through the checklists and socio-demographic and obstetric history data were collected from their mothers through face-to-face interviews after written informed consent had been obtained. Data was collected by 5 BSc female nurses after they were provided with a two-days training on the data collection tools and the supervision was done by the researcher.

### Data quality control

The instrument of data collection was pretested in neonates who were admitted to Zewditu Memorial Hospital and who were not included in the study. After the pre-test, necessary modifications were made accordingly before actual data collection was performed. Data was collected after training was given for data collectors on how to approach and interview participants and how to review charts. In the fieldwork, the principal investigator was closely following the day-to-day data collection process and ensured completeness and internal consistency of the collected data.

### Data processing and analysis

The collected data were checked for completeness and coded manually first and entered into EPI info 7.2 software, and then, were exported to SPSS version 23 software for data cleaning, coding, and analyses.

Descriptive statistics like frequencies with percentage and mean with standard deviation were used to describe the study population. Bi-variable and multivariable logistic regression analyses were performed to test the association. Variables that had a P-value of 0.2 and less were considered for the multivariable analysis. The odds ratio with 95% CI was considered to determine the presence of an association between the dependent and independent variables.

## Results

### Socio-demographic characteristics of participants

A total of 577 neonates who have mothers were included in this study. No participants met the exclusion criteria. Seven of them refused and the final number of study participants was 570, which makes the response rate 98.78%. The majority of participants 531(93.2%) were from the urban area, and the rest 39 (6.8%) were from the rural area. The mean age of participants was 26 0.66 with SD = 4.08. The largest proportion of age distribution was found between 25 to 29 years (252 (44.2%). Of all mothers involved in this study, 537(94.2%) were married. About 14.9% of mothers were none educated (Table 1).

**Table 1:** Socio-demographic characteristics of mothers whose neonates were admitted to NICU at Gandhi Memorial Hospital from Nov 1 to December 31, 2019, Addis Ababa, Ethiopia (*n* = 570)

Variables	Category	Frequency	Percentage (%)
Age (years)	≤ 24	191	33.5
	25–29	252	44.2
	30–34	98	17.2
	≥ 35	29	5.1
Educational Status	None educated	85	14.9
	Primary education	117	20.5
	Secondary education	169	29.7
	Certificate and above	199	34.9
Marital Status	Unmarried	30	5.3
	Married	537	94.2
	Separated	3	0.5
Religion	Orthodox	338	59.3
	Muslim	126	22.1
	Protestant	103	18.1
	Catholic	3	0.5
Occupation	Government	132	23.2
	Private	294	51.6
	Housewife	142	24.9
	Student	2	0.3
Monthly Family income in USA dollar	≤ 60$	143	25.1
	60.02$-100$ -	196	34.4
	100.02$-140$-	123	21.6
	> 140$	108	18.9
Residence	Urban	531	93.2
	Rural	39	6.8

#### Reproductive and obstetrics related characteristics

Among all participants, 169 (29.6%) of the mothers had an abortion history, 308 (54%) were family planning users, and 540 (94.7%) had ANC follow-up. The majority of the participants 531 (93.2%)) had taken TT immunization. Concerning in initiation of breastfeeding, 503 (88.2%) mothers initiated breastfeeding within the first hour of birth. About 26.1% of mothers had a premature rupture of membrane greater than 12 h before delivery. The majority of the mothers, 327 (57.4%) had a gestational age between 37 and 42 weeks (inclusive). Regarding the mode of delivery, 330 (57.9%) of the mothers had a spontaneous vaginal delivery, 223 (39.1%) had a Cesarean section, and the rest 17 (3%) had instrumental delivery (Table 2).

**Table 2:** Maternal Reproductive and Obstetrics characteristics of mothers whose neonates were admitted to NICU at Gandhi Memorial Hospital from Nov 1 to December 31, 2019, Addis Ababa, Ethiopia (*n* = 570)

Variable	Response	Frequency	(%)
Abortion	Yes	169	29.6
	No	401	70.4
Still Birth	Yes	5	0.9
	No	565	99.1
Family planning use	Yes	308	54
	No	262	46
ANC Follow Up	Yes	540	94.7
	No	30	5.3
ANC visit time	Incomplete(not regular)	147	25.8
	4thcomplete(Regular follow up)	423	74.2
TT immunization	Yes	531	93.2
	No	39	6.8
TT immunization dose	Not immunized	13	2.3
	≤ 2 dose	417	73.2
	> 2 dose	140	24.6
Age at first birth	15–19	33	5.8
	29–24	336	58.9
	25–29	184	32.3
	30–34	13	2.3
	35–39	4	0.7
Birth interval	First Birth	296	52
	< 2yrs	96	16.8
	2–4	147	25.8
	> 4 yrs	31	5.4
Type of pregnancy	Single	549	96.3
	Multiple	21	3.7
Initiation of breastfeeding	Yes	503	88.2
	No	67	11.8
Pregnancy-related disease	Yes	119	20.9
	No	451	79.1
Preeclampsia	Yes	112	19.6
	No	458	80.4
Diabetic Mellitus	Yes	2	0.4
	No	568	99.6
Urinary tract infection	Yes	22	3.9
	No	548	96.1
Preeclampsia and UTI	Yes	15	12.6
Diabetic Mellitus and preeclampsia	yes	1	0.84
Premature Rupture of membrane	Yes	218	38.2
	No	352	61.8
Prolonged PROM	No	421	73.9
	Yes	149	26.1
Gestational Age	< 37 wks	186	32.6
	≥ 37-42wks	327	57.4
	> 42 wks	17	3.0
	Unknown	40	7.0
Mode of Delivery	SVD	330	57.9
	Instrumental	17	3.0
	C/S	223	39.1

#### Health service care related characteristics

The majority of the mothers, 558 (97.9%), get a skilled birth attendant. In terms of delivery place, 558 (97.9%) were delivered at health facilities including health centers and hospitals, and 12(2.1%) were delivered at home.

#### Neonatal related characteristics

More than half, 347 (60.9%), of neonates admitted to NICU in the study period were male. The majority 478 (83.8%) admitted when they were less than one day old. Most, 372(65.3%), neonates weighed more than 2,500 gm at admission. According to the APGAR measure of the neonates, most, 296 (52%) were having an APGAR score between 7 & 10 in the 1^st^ minute. On the other hand, the APGAR 5^th^ score of the majority, 424 (74.4%), of the neonates was also found between 7 & 10 (Table 3).

**Table 3:** Characteristics of neonates admitted to NICU at Gandhi Memorial Hospital from Nov 1 to December 31, 2019, Addis Ababa, Ethiopia (*n* = 570)

Variable	Category	Frequency	Percentage (%)
Sex of the baby	Male	347	60.9
	Female	223	39.1
Age at admission	≤ 1 day	478	83.8
	2–7 days	91	16
	8–28 days	1	0.2
Birth weight at admission	< 2500 g	198	34.7
	≥ 2500 g	372	65.3
APGAR score at 1^st^ minute	≥ 7–10	296	52
	4–6	161	28.2
	0–3	93	16.3
	Unknown	20	3.5
APGAR score at 5^th^ minute	≥ 7–10	424	74.4
	4–6	80	14
	0–3	46	8.1
	Unknown	20	3.5

#### Medical diagnosis and causes of neonatal death

**Fig. 1:**
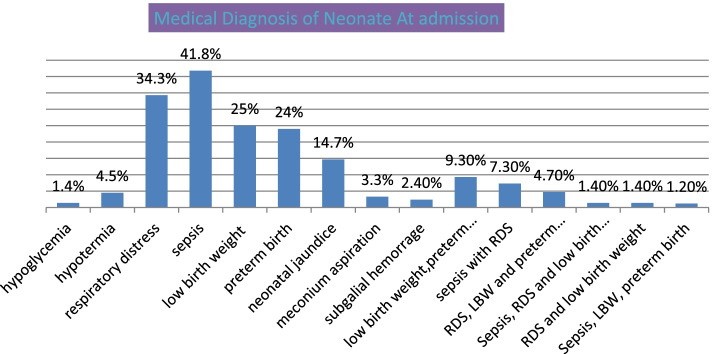
Medical diagnosis of neonates admitted to NICU at Gandhi Memorial Hospital from Nov 1– Dec 31, 2019, Addis Ababa, Ethiopia. (*n* = 570). Key RDS = Respiratory Distress, LBW = Low Birth Weight

There were different medical diagnoses made for the neonates during admission to NICU. The most prevalent cause of neonatal admission was sepsis (41.8%), respiratory distress syndrome (RDS) (34.3%), low birth weight (25%), and preterm birth (24%) (Fig. 1).

#### Prevalence and cause of neonatal mortality

Out of all neonates included in this study, 44(7.7%) (95% CI: 5.7, 9.8) neonates died from November 1 to December 31, 2019. Almost half (45.5%) of the neonates died because of respiratory distress which is followed by prematurity which accounts for 27.3%, sepsis which accounts for 11.4%, cardiac arrest for 9.1%, and congested heart failure for 6.8%.

#### Factors associated with neonatal mortality

Among the variables included in the multivariable analysis, maternal educational status, PROM, and birth weight had a significant association with neonatal mortality. The odds of having neonatal mortality for neonates from non-educated mothers is 3.37 ((AOR: 3.37; 95% CI: 1.02, 11.20) times higher compared to neonates from educated mothers The odds of having neonatal mortality for neonates of Mothers with prolonged PROM is 5.59 ((AOR: 5.59; 95% CI:1.05, 29.76) times higher than neonates of mothers not having prolonged PROM. On the other hand, the odds of neonatal mortality among neonates with a birth weight less than 2500gm is 3 (( AOR: 3.23;95% CI:1.17,8.90) times higher compared to neonates whose birth weight is 2500gm and above (Table 4).

**Table 4:** Bi variable and multivariate logistic regression analyses for neonatal mortality among neonates who were admitted to Gandhi memorial hospital Addis Ababa, 2019 (*n* = 570)

Variable	Category	Neonatal Death		COR(95%CI)	AOR(95%CI)
		Died	Not died		
Age of Mother	≤ 24	8(4.2%)	183(95.8%)	0.17(0.05,0.526)	0.21(0.03,1.27)
	25–29	19(7.5%)	233(92.5%)	0.31(0.11,0.86)	0.55(0.12,2.64)
	30–34	11(11.2%	87(88.8%)	0.49(0.16,1.450)	0.30(0.05,1.90)
	≥ 35	6(20.7%)	23(79.3%)	1	1
Educational Status	No education	17(20%)	68(80%)	4.27(1.91,9.581)	3.37(1.02,11.20)*
	Primary Education	5(4.3%)	112(95.7%)	0.76(0.26,2.25)	0.96(0.20,4.52)
	Secondary Education	11(6.5%)	158(93.5%)	1.19(0.50,2.82)	0.54(0.12,2.31)
	Above Secondary Education	11(5.5%)	188(94.5%)	1	1
Pregnancy-Related Disease	Yes	15(12.6%)	104(87.4%)	2.10(1.09,4.06)	0.78(0.20,3.08)
	No	29(6.4%)	422(93.6%)	1	1
Prolonged PROM	No	21(14%)	400(86%)	1	1
	Yes	23(15.4%)	126(84.6%)	5.66(1.29,24.77)	5.59(1.05,29.76)*
Birth Weight	< 2500	26(13.1%)	172(86.9%)	2.97(1.59,5.57)	3.23(1.17,8.91)*
	≥ 2500	18(4.8%)	354(95.2%)	1	1
Hypoglycemia	Yes	3(37.5%)	5(62.5%)	7.62(1.76,33.04)	1.40(0.11,17.35)
	No	41(7.3%)	521(92.7%)	1	1
Hypothermia	Yes	5(19.2%)	21(80.8%)	3.08(1.10,8.62)	2.53(0.49,13.21)
	No	39(7.2%)	505(92.8%)	1	1
Respiratory Distress	Yes	28(14.3%)	168(85.7%)	3.73(1.96,7.08)	2.24 (0.74,6.83)
	No	16(4.3%)	358(95.7%)	1	1
Preterm Birth	Yes	17(12.4%)	120(87.6%)	2.13(1.12,4.04)	1.74(0.33,9.03)
	No	27(6.2%)	406(93.8%)	1	1
Apgar 1^st^	≥ 7–10	0(0.0%)	296(100%)	1	1
	4–6	8(5%)	153(95%)	4.07(1.67,9.95)	1.56(0.40,6.04)
	0–3	27(29%)	66(71%)	6.88(2.70,17.50)	0.60(0.02,19.30)
	Unknown	9(45%)	11(55%)	10.64(3.94,28.70)	2.25(0.25,20.00)
Apgar 5^th^	≥ 7–10	0(0.0%)	424(100%)	1	1
	4–6	27(33.8%)	53(66.2%)	10.04(4.90,20.60)	2.66(0.71,9.88)
	0–3	8(17.4)	38(82.6)	3.23(1.12,9.31)	2.18(0.47,10.0)
	Unknown	9(45%)	11(55%)	9.22(2.27,37.66)	9.65(0.93,100.5)

1 = Reference, * = *P* < 0.05, *PROM* Premature rapture of membrane

**Bold = significant categories**

## Discussion

This study assesses the prevalence and associated factors of neonatal mortality among newborns admitted to NICU in Gandhi memorial hospital. The prevalence of neonatal mortality was 7.7 (95% CI: 5.7, 9.8). Being a Non-educated mother, prolonged PROM and low birth weight at delivery were significantly associated with neonatal mortality.

The prevalence of neonatal mortality in this study was 7.7 (95% CI: 5.7, 9.8). It was lower than a study conducted in Bahir Dar (13.29) [14], Jimma (35.5%) [15], India (26.26) [16], and Congo (47%) [17]. The possible explanation for the difference can be the difference in the study design, and study setting. For example, evidence from Jimma was generated by a community-based prospective follow-up study [15]. Being community-based will help the investigators to trace the hidden mortality that occurs without getting medical care at home and being a prospective follow-up study will increase the chance of getting appropriate evidence over a long period [15, 16]. Evidence from India was from a prospective follow-up study on neonates of 24hs old admitted at NICU [18]. It is obvious that the highest percentage of neonatal mortality occurs within the 1^st^ 24 h of life and being admitted exacerbates the rate of mortality. In the India study, the leading causes of mortality were higher (low birth weight babies were 63.5% and respiratory distress was present in 47.2% of neonates) [18] compared to the current study.

Our finding is almost comparable with a study conducted in Northern Ethiopian, Ayder referral hospital (6.6%) [19].

But it was higher than a study conducted in Zambia (3.4%) [20]. This variation in the result of neonatal mortality might be due to the difference in the study population. In our study, the study populations were all neonates admitted to ICU whereas, in Zambia, they were all live births. Admitted neonates have a high chance of being died as compared to non-admitted and relatively healthy neonates.

Maternal education was one of the factors associated with neonatal mortality. Newborns whose mothers were illiterate or non-educated were almost more than threefold times higher risk of having neonatal mortality when compared with educated or literate mothers. This finding is in line with a study conducted in Zambia and a systematic review conducted in Ethiopia that infants born to mothers with lower education were associated with increased odds of dying [20, 21]. The above systematic review showed that attending primary education was associated with a 28% reduction and attending secondary education and above was associated with a 45% reduction in the odds of infant mortality compared to those infants born to illiterate mothers [21] The possible explanation for this could be, Education levels can influence newborn survival by affecting reproductive behavior and improving mothers' health-care seeking behavior in areas such as contraception, nutrition, hygiene, preventative care, and disease treatment [22]. Educated mothers also provide better care for themselves during pregnancy and for their children during the most vulnerable stages of their lives than non-educated mothers [23] which can enhance the survival of neonates.

In this finding, mothers having prolonged PROM have higher odds of neonatal mortality compared to mothers who had no prolonged PROM. This finding is consistent with studies conducted in Jimma and Arbaminch that premature and prolonged rupture of membrane before the onset of labor > 12 h had increased the likelihood of neonatal death [9, 24]. This might be as the time of rupture of membrane increases, the risk of respiratory distress and sepsis for a neonate with a PROM mother will increase [25]. PPROM is associated with neonatal death due to prematurity complications, infection, and pulmonary hypoplasia [26]. Evidence from the International federation of gynecology and obstetrics (FIGO) shows, PPROM can contribute for about 20% of all perinatal death. It also leads to significant perinatal morbidity associated with prematurity such as respiratory distress syndrome, neonatal sepsis, umbilical cord prolapse, placental abruption, and fetal death [27].

The other finding of this study was low birth weight. The odds of neonatal mortality among birth weight less than 2,500 g were almost threefold higher as compared to birth weight greater than 2,500 g. This finding is supported by studies conducted in Bangladesh [28] Nigeria [29] and Assosa [30] Zone that found neonates born at low birth weights were at a higher high risk of death compared to normal birth weight neonates.. This may be the case in that babies with low birth weight might be unable to cope-up with a new environment and possible infections and as a result, could be at risk of neonatal death as compared to normal birth weight. Another possible explanation can be, that small size neonates were highly susceptible to different infections due to having low immunity defense [30].

### Limitation of the study

Since this study was conducted in a hospital sample that may be different from the general population; it may not be inferred to neonates who are dying out of the health institution.

## Conclusion

The prevalence of neonatal mortality at Gandhi memorial hospital was generally high. As our finding revealed, neonates who are underweight and whose mothers have no formal education as well as have prolonged PROM have higher odds of neonatal mortality. Thus, clinicians, policymakers, and program managers should give special attention to neonates of none educated mothers, mothers with prolonged PROM, and neonates with low birth weight.

## Supplementary Information

**Additional file 1.**

## Data Availability

The datasets generated and/or analyzed during the current study are available from the corresponding author and are being provided to the journal in the related file section.

## Abbreviations

ANC: Antenatal Care
COR: Crude odds ratio
AOR: Adjusted Odd Ratio
APGAR: Appearance, Pulse, Grimace, Activity, and Respiration
CI: Confidence Interval
NICU: Neonatal Intensive Care Unit
NMR: Neonatal Mortality Rate
OAU: Organization of African Union
OR: Odds Ratio
PROM: Premature Rupture of Membrane
PPROM: Prolonged Premature Rupture of Membrane
SVD: Spontaneous vaginal delivery
CS: Cesarean section
SD: Standard deviation
TT: Tetanus toxoid
SPSS: Statistical Package for the Social Science
