# Supplementary figures and images for: Neonatal mortality and associated factors among neonates admitted to neonatal intensive care unit of Gandhi memorial hospital in Addis Ababa, Ethiopia, 2019
Source: BMC Pediatr. 2022 May 12;22:266. doi: 10.1186/s12887-022-03339-6 (PMC9097131; doi:10.1186/s12887-022-03339-6)

## Annex 1 Ethical clearance


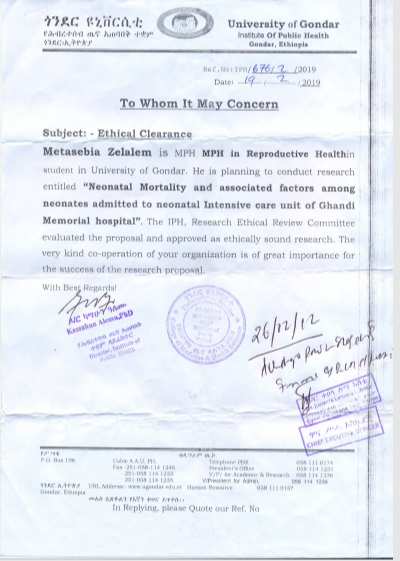

Supplement: Supplementary file 1 — Additional file 1. [file 12887_2022_3339_MOESM1_ESM.docx]
